# Supplementary figures and images for: Escherichia coli β-clamp slows down DNA polymerase I dependent nick translation while accelerating ligation
Source: PLoS One. 2018 Jun 20;13(6):e0199559. doi: 10.1371/journal.pone.0199559 (PMC6010275; doi:10.1371/journal.pone.0199559)

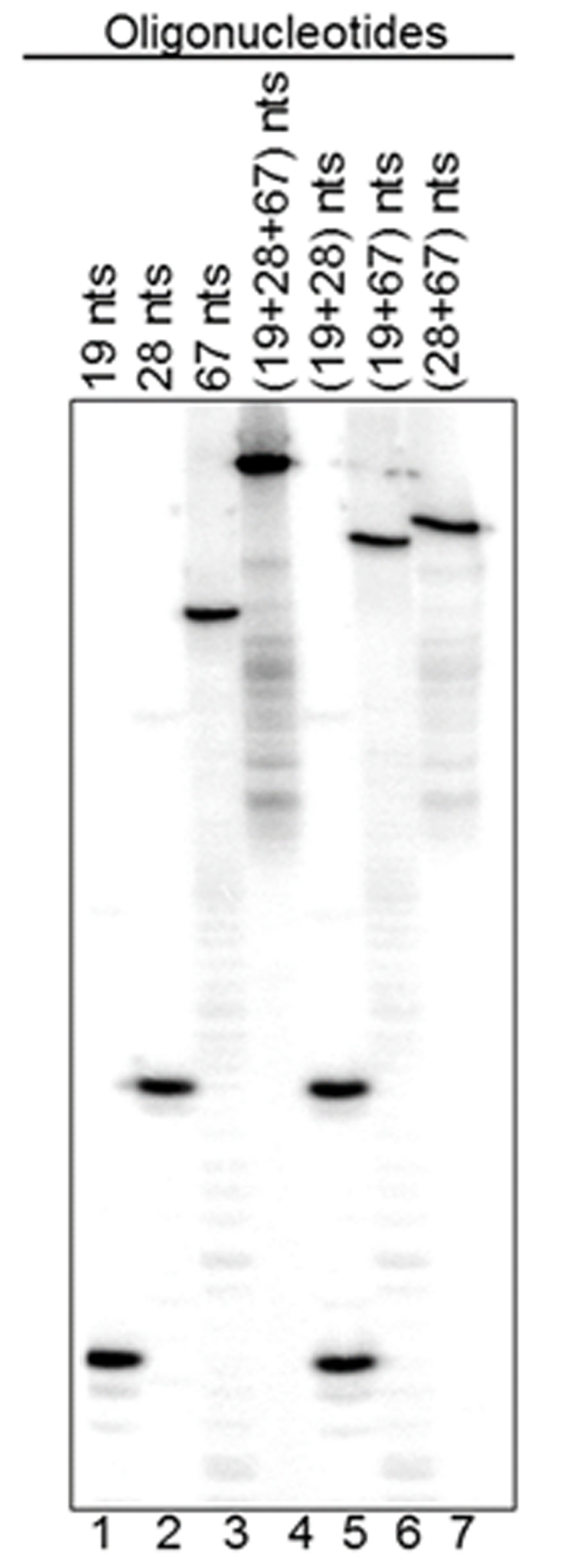

Supplement: S1 Fig — The native gel shows the separation of 19 nucleotides (lane 1), 28 nucleotides and 67 nucleotides (lane 3) long oligonucleotides. When they mixed in an annealing reaction, the individual oligonucleotides disappeared from their original position and gave rise to a higher band (Lane 4), suggesting that annealing was 100% efficient. However, non-complementary oligonucleotides (19 and 28 bases) did not found to be annealed (lane 5). On the other hand, the 67 bases long oligonucleotide, which has complementary regions for both 19 and 28 bases long oligonucleotides, has also completely annealed to these 19 and 28 bases oligonucleotides (lanes 6 and 7, respectively). (TIF) [file pone.0199559.s001.tif]

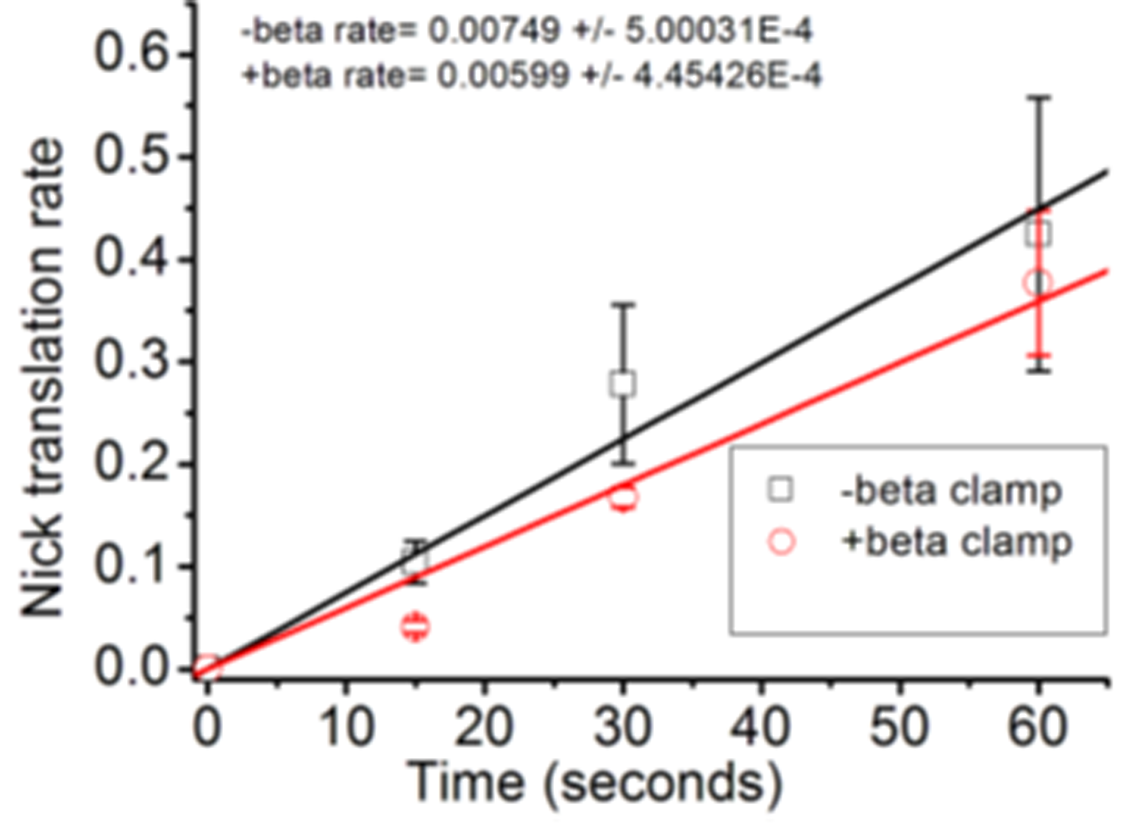

Supplement: S2 Fig — The fraction of the initial counts that appeared at 67 nucleotide position were plotted. The rate of the nick translation through RNA substrate was calculated from the fitted curve shown above (also see S1 Text). The negligible difference between the rate of nick translation in the presence and absence of β-clamp, suggests that β-clamp has no influence on nick translation through RNA substrates. (TIF) [file pone.0199559.s002.tif]

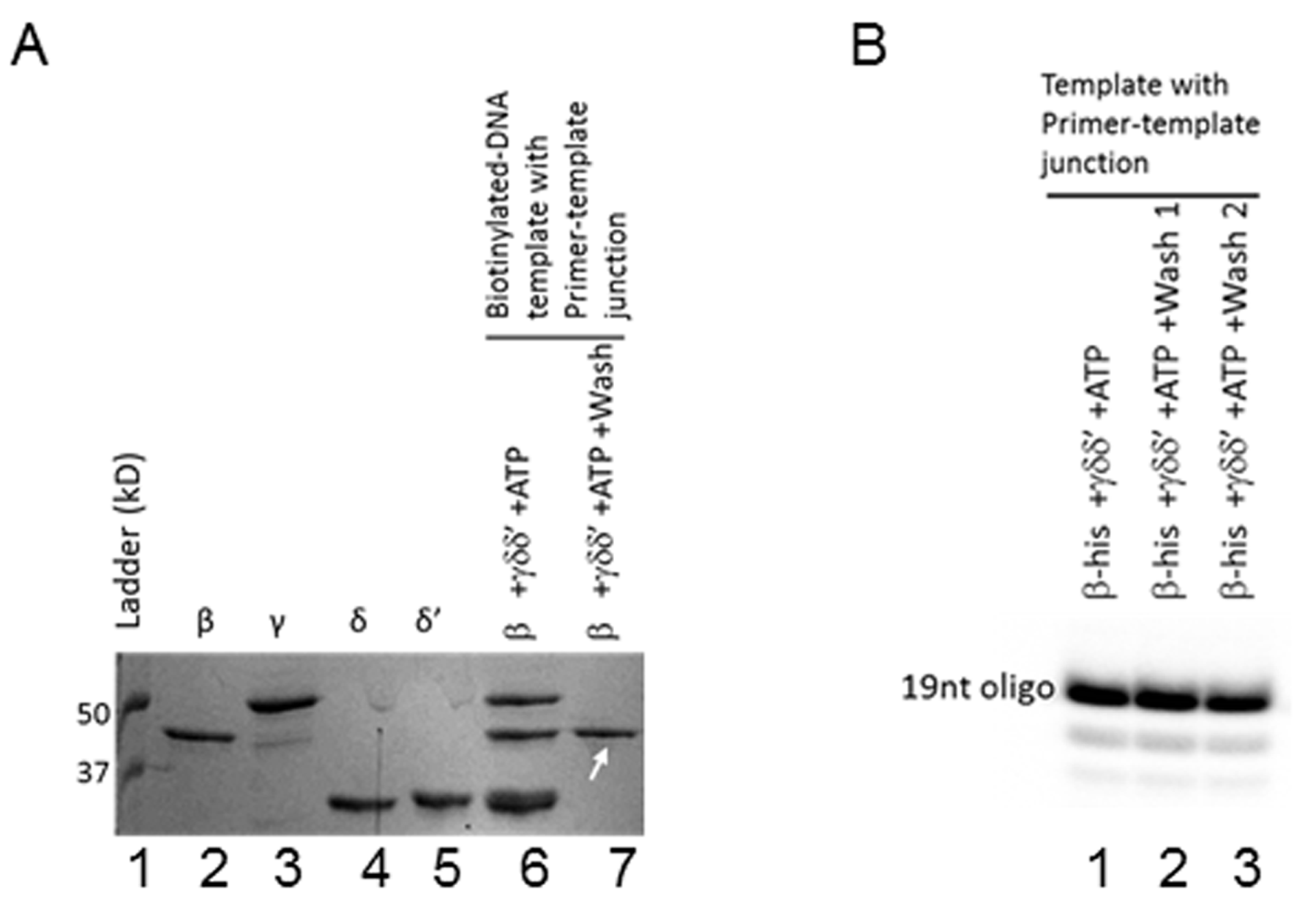

Supplement: S3 Fig — (A) The lanes 2–5 are showing purified individual proteins that are used in the pull down assay. The streptavidin-bound primed biotinylated template could pull-down β-clamp (marked by white arrow), while clamp loader proteins washed away (compare unwashed and washed lanes 6 and 7, respectively). (B) The his-tag β clamp pull down primed template, as indicated by the pulled down radiolabeled 19nt long oligonucleotide, which was the integral part of the template. (TIF) [file pone.0199559.s003.tif]

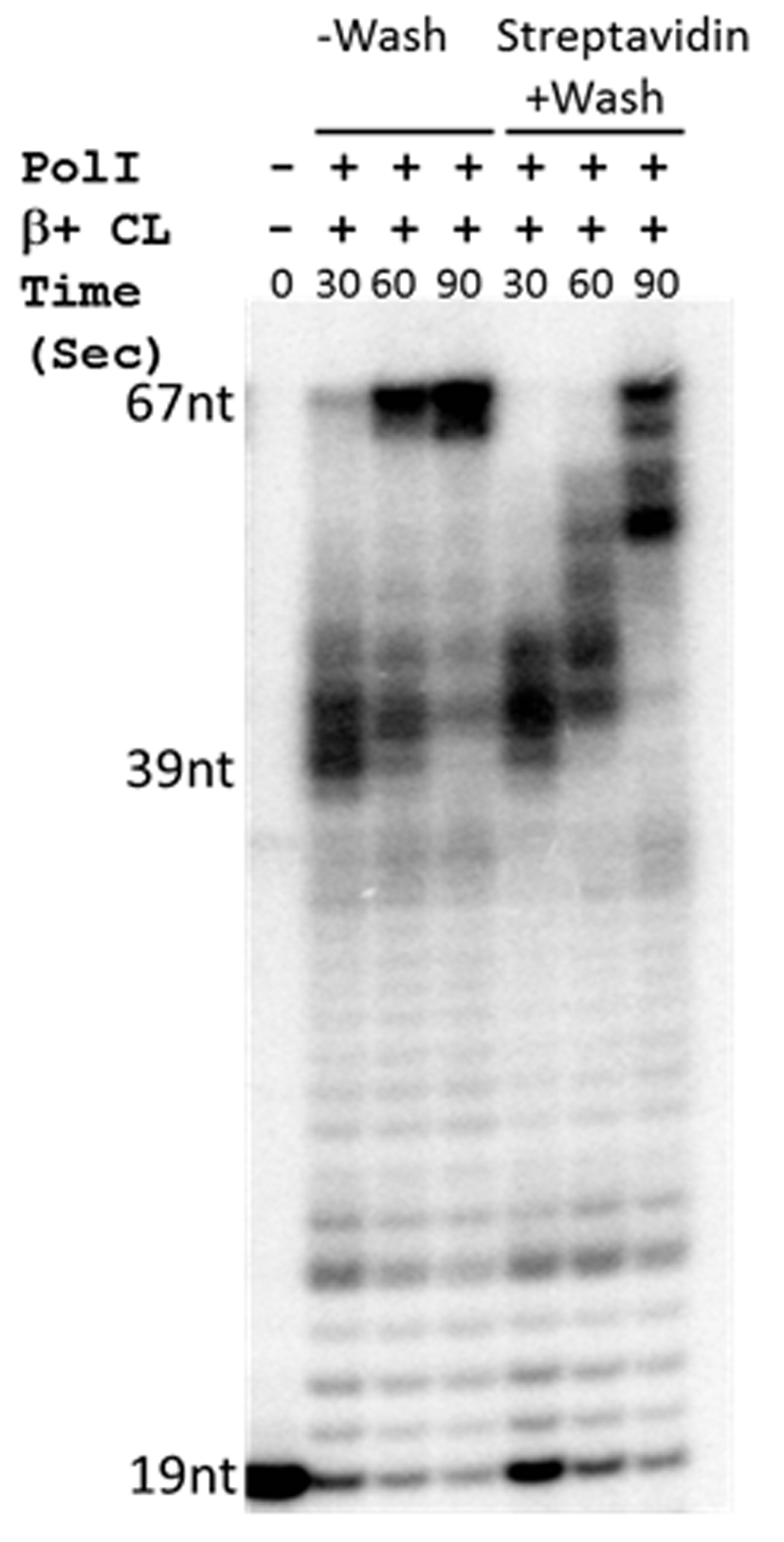

Supplement: S4 Fig — The urea denaturing gel indicating that washing the streptavidin-bound reaction products, which washes out the clamp loader protein, but retains β clamp (S3 Fig), generates similar pauses (+wash lanes) as observed for standard reaction (-wash lanes) performed in the presence of β clamp loader proteins. (TIF) [file pone.0199559.s004.tif]

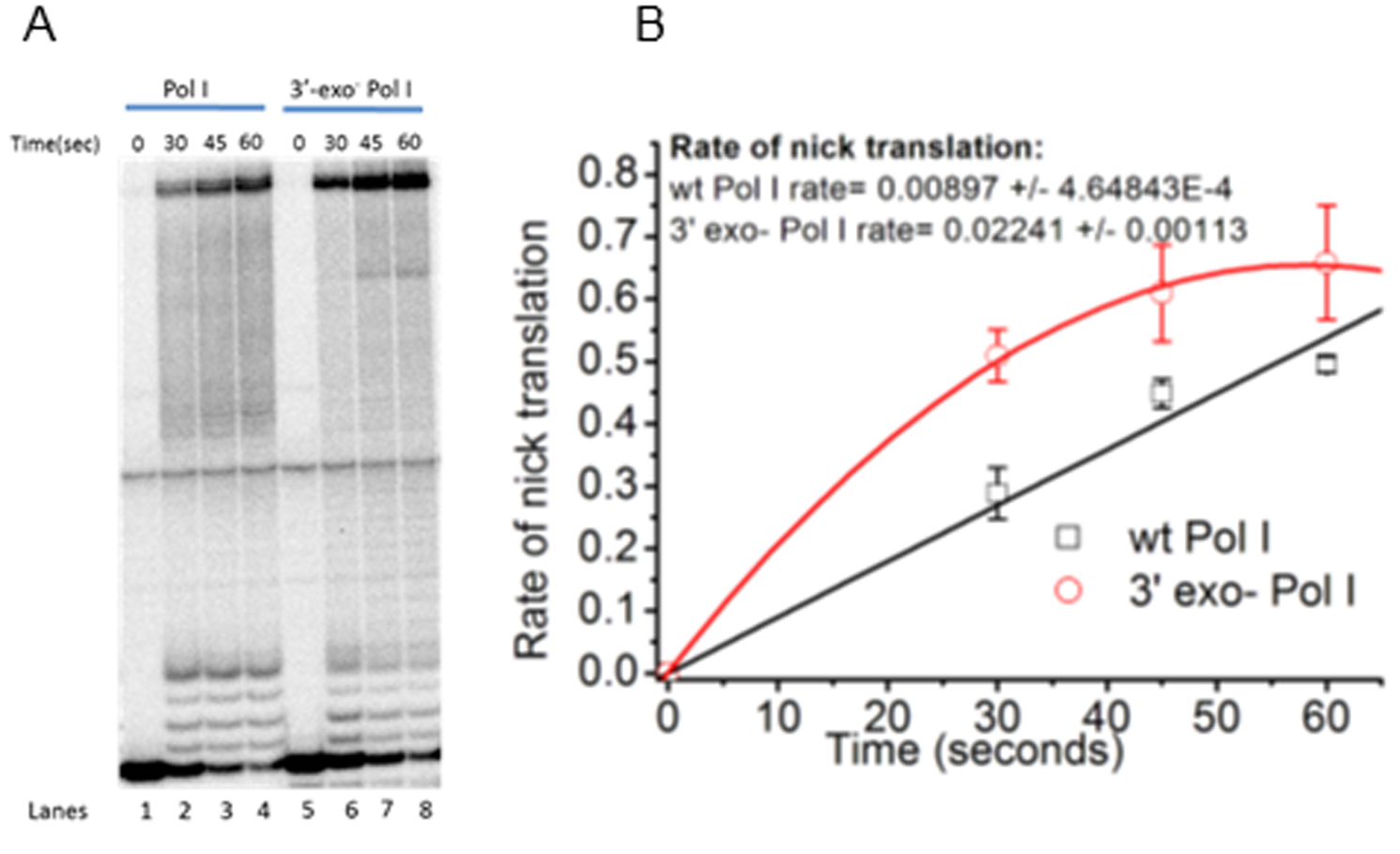

Supplement: S5 Fig — (A) The autoradiogram represents nick translation product formation at three different time points by Pol I and 3’-exo- Pol I. (B) The rate of nick translation was calculated as described in S1 Text, from three independent autoradiograms and plotted. The initial rate of nick translation was found to be faster in case of 3’-exo- Pol I. (TIF) [file pone.0199559.s005.tif]

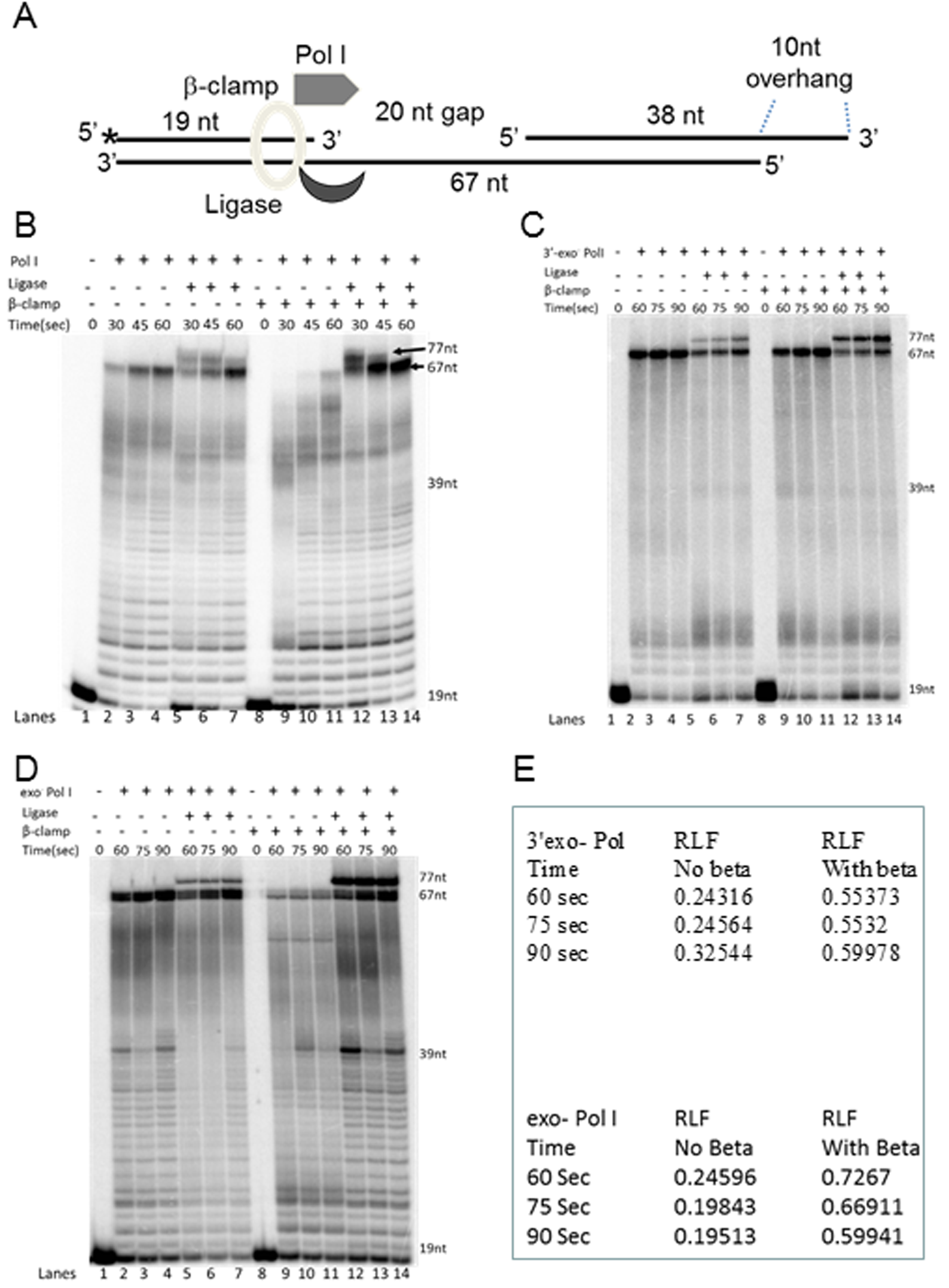

Supplement: S6 Fig — (A). A new template was assembled using 67 bases, 5’-phosphorylated 38 bases and 5’-radiolabelled (asterisk) 19 bases oligonucleotides, so that a 10 nucleotide long 3’ overhang was generated as shown. (B) The autoradiogram represents Pol I mediated nick translation-coupled ligation in the presence or absence of β-clamp. Visibly, 77 nucleotide ligation product that appears at lower time points, degraded quickly due to 3’ exonuclease activity of intact Pol I. (C, D) The representative autoradiograms show that 3’-exo- Pol I and exo- Pol I produced stable nick translation-coupled ligation products in the presence or absence of β-clamp in three different time points. (E) RLFs calculated at different time points in the presence or absence of β-clamp from panels S6C and S6D Fig are shown. (TIF) [file pone.0199559.s006.tif]

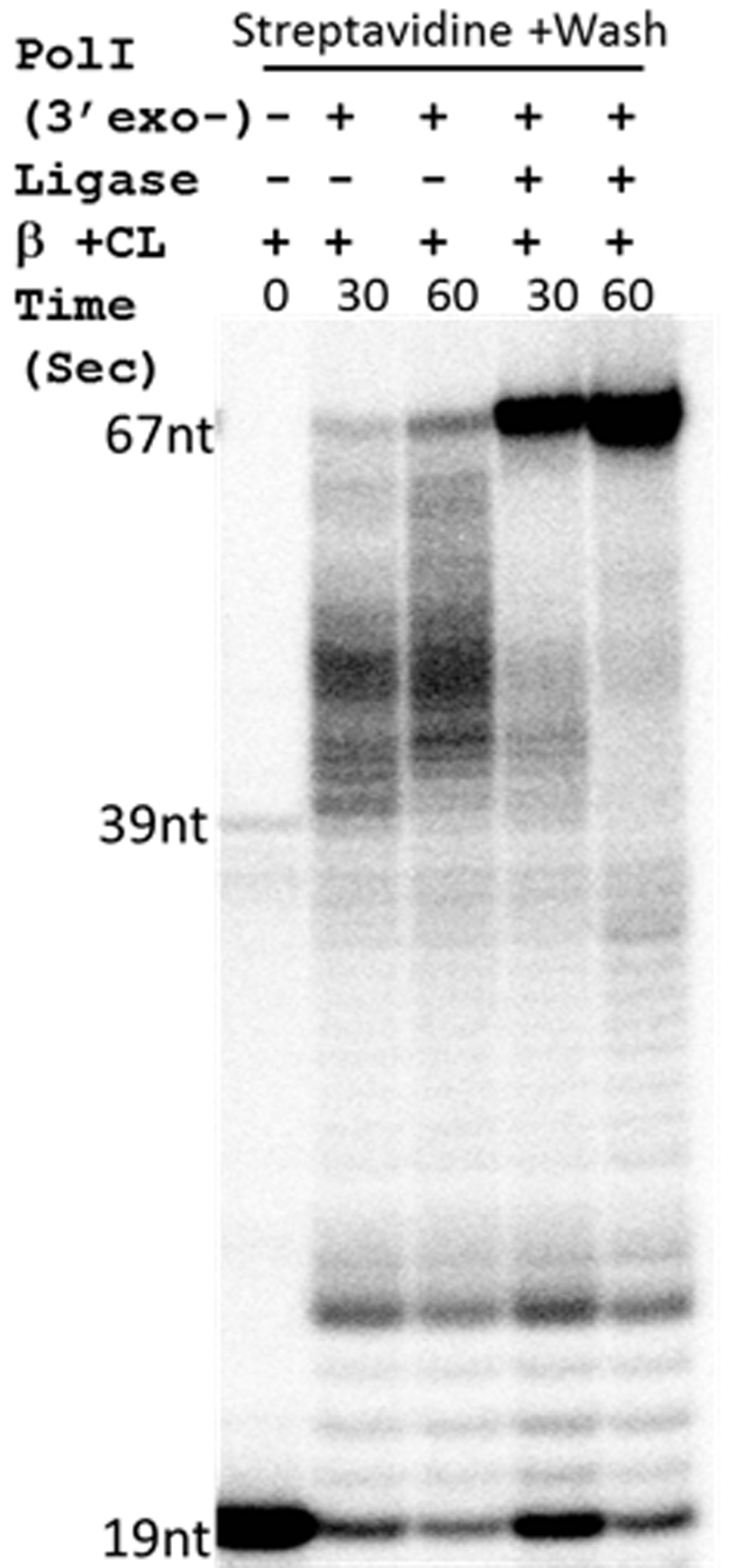

Supplement: S7 Fig — The nick translation coupled ligation assay was performed on streptavidin beads to remove clamp loader proteins after loading of the β-clamp. Addition of 3’ exo- Pol I and dNTPs exhibited pausing at the 39th and downstream locations at two different time points. Addition of the Pol I and ligase efficiently produces 67nt products, suggesting that in the absence of clamp loader complex the nick translation coupled ligation reactions are similar as demonstrated in Fig 1D. (TIF) [file pone.0199559.s007.tif]

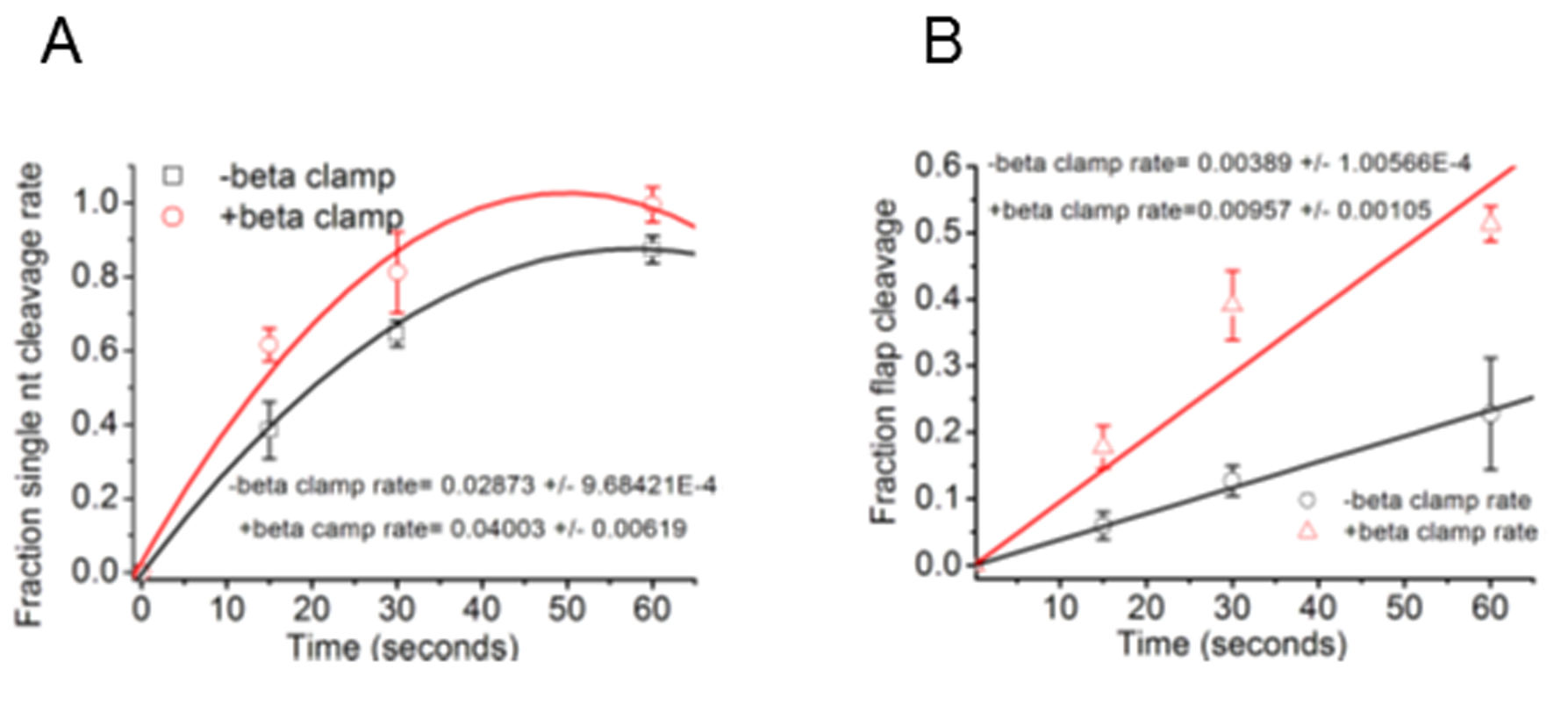

Supplement: S8 Fig — (A). Single nucleotide cleavage was shown in Fig 4C. The single nucleotide fraction cleavage values at different time points were plotted to calculate the rate. The presence of β-clamp was found to modestly increase the single nucleotide cleavage. (B) Similarly, flap cleavage was shown in Fig 4D and the flap fraction cleavage values at different time points were plotted to calculate the flap fraction cleavage rates. Interestingly, β-clamp greatly enhanced the flap cleavage rate. (TIF) [file pone.0199559.s008.tif]

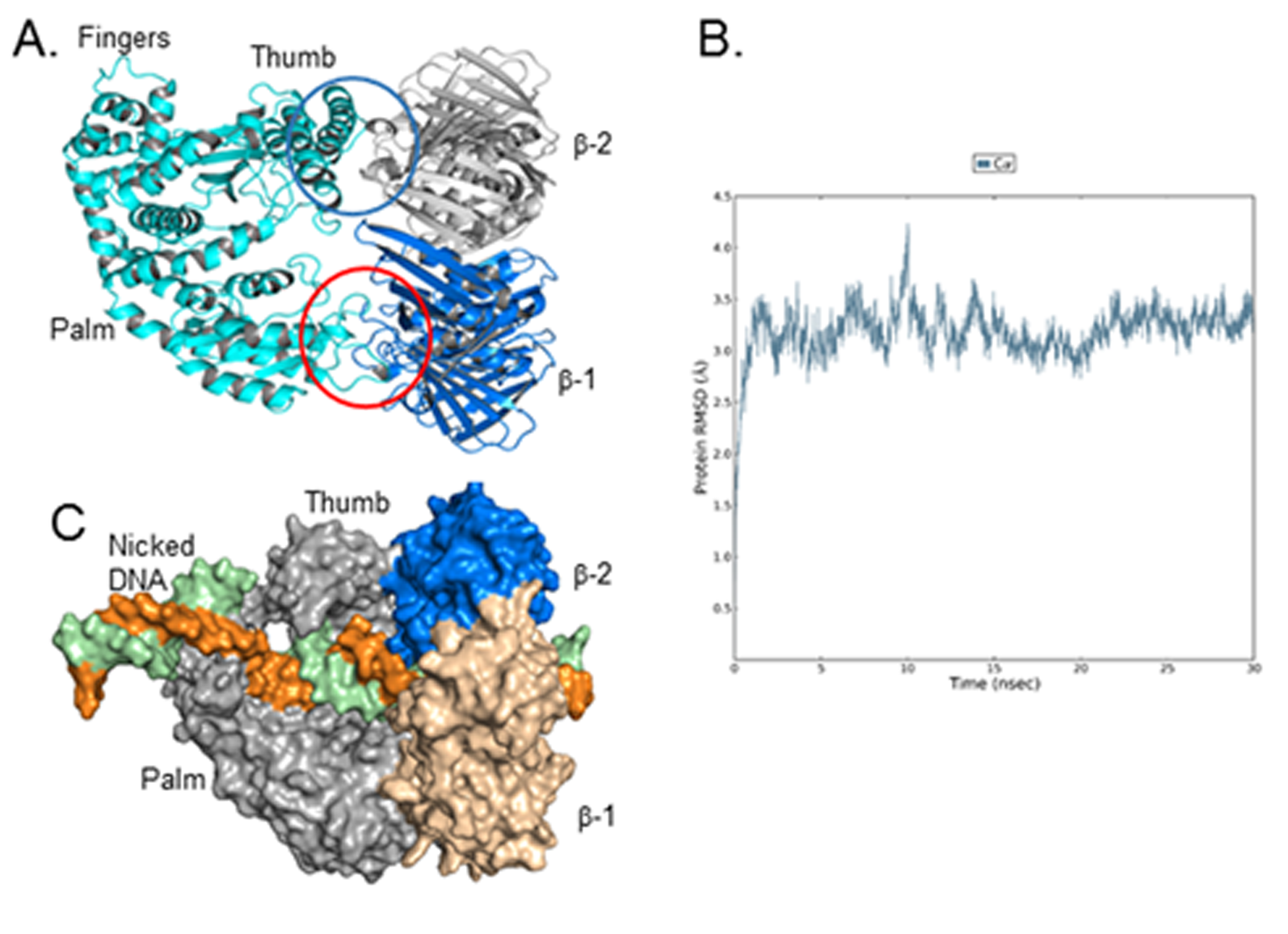

Supplement: S9 Fig — (A) Cartoon representation of the docked model of Klenow (cyan) /β-clamp (gray and blue) protein complex. The possible protein-protein interaction interfaces are encircled in red and blue. (B) Molecular dynamics simulations for the docked Klenow/β-clamp protein-protein complex. (C) Surface representation of the modelled Klenow (grey) /β-clamp (yellow and blue)/ DNA (orange and green) ternary complex. (TIF) [file pone.0199559.s009.tif]

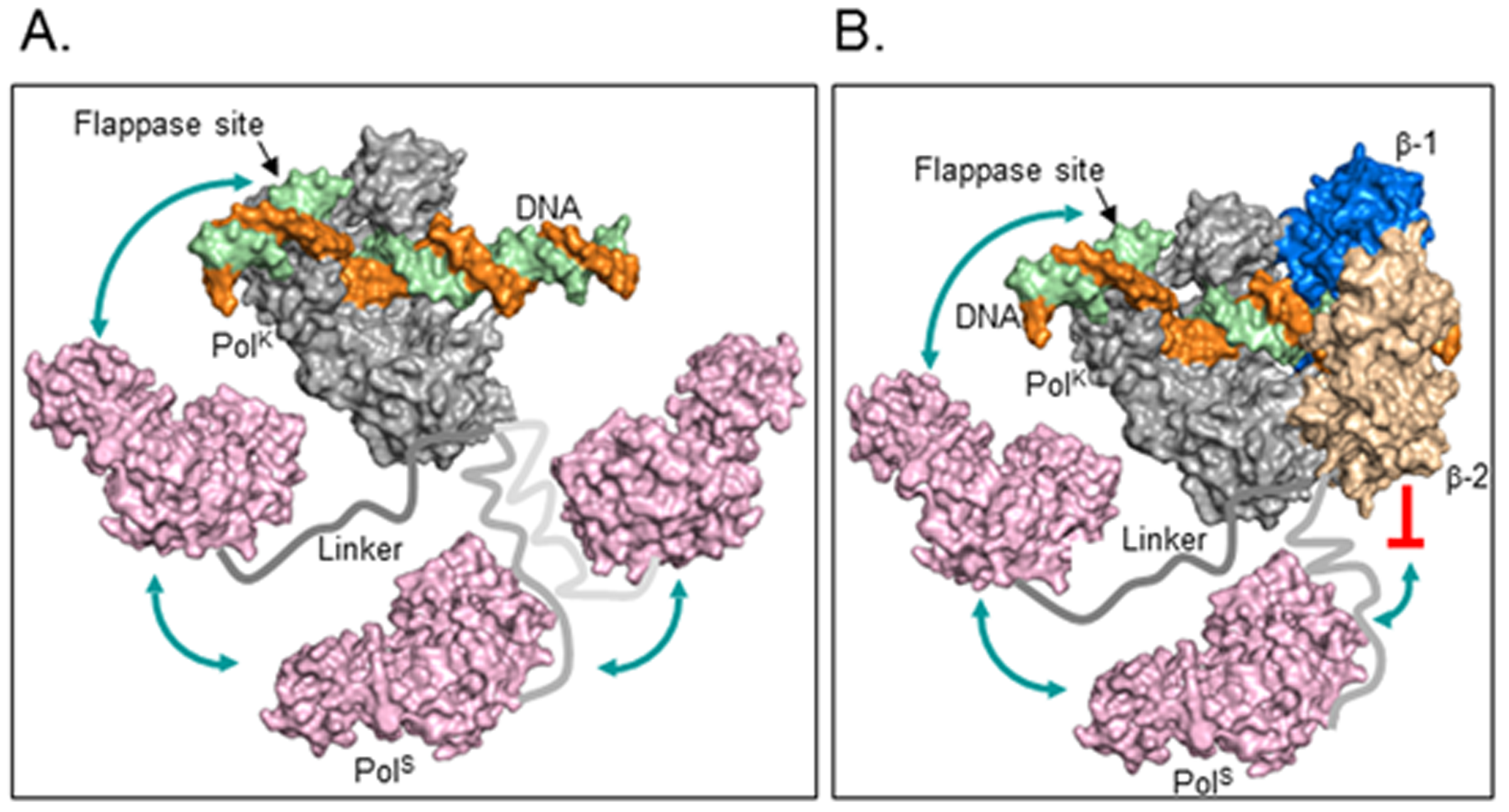

Supplement: S10 Fig — The structural model of E. coli small domain of Pol I (light pink) was built using Prime module in the Schrodinger software suite using 1BGX as the template. The models were manually placed near the Klenow/nicked DNA (A) and Klenow/ β-clamp/nicked DNA (B) models using PyMOL. The predicted flexible linker region connecting small domain and Klenow fragment has been shown by undulating lines in different grey shades. The conformational space available in β-clamp-bound and β-clamp free Klenow/nicked DNA structures are shown by multiple copies of small domains. From this model, we hypothesize that the presence of β- clamp could sterically restrict (shown by red T blocker) the conformational freedom of small domain hence increasing the chance of accessing 5’ exonuclease site. (TIF) [file pone.0199559.s010.tif]
