# Supplementary material for: Escherichia coli β-clamp slows down DNA polymerase I dependent nick translation while accelerating ligation
Source: PLoS One. 2018 Jun 20;13(6):e0199559. doi: 10.1371/journal.pone.0199559 (PMC6010275; doi:10.1371/journal.pone.0199559)
Supplement: S1 Text — (DOCX) [file pone.0199559.s011.docx]

**S1 TEXT**

***Escherichia coli* β-clamp slows down DNA polymerase I dependent nick translation while accelerating ligation**

Amit Bhardwaj^1^, Debarghya Ghose^1^, Krishan Gopal Thakur^1^ and Dipak Dutta^1*^

CSIR-Institute of Microbial Technology, Chandigarh, India^1^

**^*^** Corresponding author

E-mail: [dutta@imtech.res.in](mailto:dutta@imtech.res.in) (DD)

**Supporting Materials and Methods**

**Purification of the histidine tag β-clamp**

PCR of the gene, *dnaN*, encoding β-clamp was performed and cloned at the *Nde*I and *Eco*RI sites of pET28a (+) vector and expressed as a histidine-tagged protein. The overexpressed protein was purified by Ni-NTA affinity chromatography.

**Making templates for control experiments**

Along with the oligonucleotides described in the main text, we used few other PAGE purified oligonucleotides. They were, 5’-biotinylated 67 bases: GGATCCCACACTCATTAAAAT TAGTCGCTAATGCATTCTAAAAGCATTCGCAACGAGAAGATAGAGG and 39 bases: TAATGCATTCTAAAAGCATTCGCAACGAGAAGATAGAGG in lengths. Using same protocol mentioned in the main text, we annealed the oligonucleotides in different combinations to get blunt end and primed template for the assays, as described below in Supplementary results section.

**RLF Quantification**

We adopted standard quantification method using Fujifilm FLA-9000 phosphor imager or Biorad PharoxFX Plus molecular imager to determine the intensity of the bands/products in the autoradiograms represented throughout the manuscript. The intrinsic photo-stimulated luminescence (PSL) or phosphor-imaging counts of the products and the background were obtained by selecting the zone of interests from the autoradiograms in triplicate. We subtracted the background counts from the product counts. Background subtracted phosphor-imaging counts of final 67 nucleotides long product, and nick-translated and nick translation-coupled ligation products were used in the formula described in the main text.

**Rate calculation for nick translation and flap cleavage**

To calculate the rate of nick translation by Pol I and 3’ exo^-^ Pol I (as shown in S5 Fig) or by Pol I through RNA substrate (as shown in Fig 1B and S2 Fig), the background-subtracted counts of the 67 nucleotides products at different time points and counts of 19 nucleotides initial substrates (eg. lanes 1 and 5 of Fig. 1B) were acquired. Fraction of the counts that appeared at 67-nucleotide position at different time points were calculated by dividing the background-subtracted counts of 67 nucleotides product with the background-subtracted counts of 19 nucleotides initial substrates. Three independent experiments were performed to calculate the fraction counts. The fraction counts were plotted using Microcal Origin software and fitted to calculate the rates of nick translation in the presence and the absence of β-clamp.

To calculate the rate of single or 10 nucleotide cleavage, the background subtracted counts of the 1 or 10 nucleotides cleavage products at different time points and counts of 28 or 38 nucleotide initial substrates (Lanes 1 and 5 of Fig. 4C and 4D) were acquired. Fraction of the counts that appeared at 1 or 10 nucleotide positions at different time points were calculated by dividing the background-subtracted counts of 1 or 10 nucleotides products with the background subtracted counts of respective initial substrates. Three independent experiments were performed to calculate the fraction counts. The fraction counts were plotted using Microcal Origin software and fitted to calculate the initial rates of cleavage in the presence and the absence of β-clamp, as shown in S8 Fig.

**Model building: protein-protein docking and molecular dynamics simulations**

Currently there is no structural information available for the Klenow /β-clamp proteinprotein complex. For docking *E. coli* Klenow and β-clamp PDB IDs 1D9D and 5FKW (structural coordinates were extracted for homodimeric β-clamp only), respectively were used as input models to perform protein-protein docking using Cluspro 2.0 server [55]. Several output models of docked Klenow at various sites on homodimeric β-clamp were generated by this software. In a few models, two regions of Klenow (IAGKGKNQLTFNQ and DPKVLHNHSEELTLRLAELE KKAH) docked well in the reported β-clamp binding pockets [56]. Interestingly, portion of the one binding region (IAGKGKNQLTFNQ) has a weak sequence similarity with predicted QL(S/D)LF consensus sequence that interacts with β-clamp binding pockets [57]. We further analyzed these models by fitting a probable DNA path through the Klenow /β-clamp protein complex. For this, information from the cryo-EM structure of the *E. coli* replicative DNA polymerase complex bound to DNA (PDB ID: 5FKW) and *Geobacillus stearothermophilus* Klenow complexed to 9 base pairs of duplex DNA (PDB ID 1L3S) were used. The structural modelling and superposition were carried out in COOT [58]. From this exercise, we selected a docked model that represented minimum strain and steric clashes between protein and DNA components, so that an overall straight path of DNA was through the β-clamp and the Klenow. To check the stability of the docked protein-protein complex, the Klenow /β-clamp complex model was subjected to 30 ns molecular dynamics simulations in the NPT ensemble using Desmond module of the Schrodinger software suite [59-61]. Briefly, structure preparation and minimization were carried out using Protein Preparation Wizard, and the missing loop region in Klenow was built using Prime module in the Schrodinger software suite [62,63]. Restrained minimization was performed using the OPLS-2005 force field [60] to converge all heavy atoms to 0.3 Å root-mean-square deviations (RMSD) of the initial model. OPLS 2005 force field was used to build aqueous biological systems, and the TIP4P model was used to simulate water molecules. The orthorhombic boundary conditions were set up with buffer of 10 Å. Electrically neutral system was created by randomly placing the minimum number of sodium and chloride ions needed to balance the system in the solvated system. The full system was simulated using the default multistep MD protocols in the Desmond module using maestro 2016-2 [58]. The simulations were carried out at 300 K with a recording interval of 30 ps. All the structural figures were made with PyMOL [64].

**Supporting results**

**β-clamp** **binds only to the primed template DNA**

Two different biotinylated templates were prepared in this case. Annealing 19, 28 and 5’-bio-67 oligonucleotides generated the biotinylated primed template with 3’-recessed end. The template at 20 nM concentration was incubated with 30 nM β-clamp, 1mM ATP and 30 nM clamp loader complex in a 100µl reaction for 2 minutes at 37°C to allow β-clamp loading in the reaction buffer mentioned in the main text. 50 µl of the mixture kept aside and other 50 µl was incubated with streptavidin beads for 3 minutes, and washed twice (300 µl each wash) and suspended in 50 µl reaction buffer. SDS loading dye was added in both of the reaction mixtures, reduced the volumes by heating and loaded on a SDS PAGE. We show that the primed biotinylated template pulled β-clamp down, while the clamp loader proteins were washed away. This observation suggest that β-clamp strongly interacts with a primed template after a clamp loading reaction (S3A Fig). This observation vindicates the earlier observations that the clamp loader has no affinity with the β-clamp and template after it loads β-clamp [9,12,47].

Next, we used his-tag β-clamp for the template pull down assay. We generated primed template annealing 39nt and radiolabeled 19nt oligonucleotides. his-tag β-clamp was incubated with the template for 2 minutes by clamp loader complex and ATP. The reaction mixture was incubated with Ni-NTA beads and was divided into three equal aliquots. First aliquot was kept intact. Second aliquot was washed once (300 µl wash) with the reaction buffer. Third aliquot was washed twice (300 µl each wash) with the reaction buffer. All of the aliquots were loaded on a urea denaturing gel to detect the radiolabeled 19 residues long oligonucleotide. We find that the 19nt oligonucleotide count was unchanged even after washing once or twice, suggesting that histidine-tagged β-clamp remains tightly bound and 100% occupied with the template (S3B Fig).

**Clamp loader proteins do not interfere with nick translation**

We prepared the primed-biotinylated DNA template and loaded β-clamp, as described above. DNA polymerase I and dNTPS were added in one-half of the reaction mix to perform nick translation assay. The other-half was incubated with streptavidin beads for 3 minutes and washed twice (300 µl each wash) with the reaction buffer to remove clamp loader proteins. Next, DNA pol I and nucleotides were added to see the extension. In both cases, we see similar inhibition in nick translation by Pol I (S4 Fig), suggesting that the presence or absence of clamp loader proteins do not determine such inhibition.

**Clamp loader proteins do not interfere with the ligation process**

The primed template was prepared annealing 19, 5’-phosphorylated 28 and 5’-biotinylated 67 residues long oligonucleotides. After β-clamp loading, the reaction mixture was incubated with streptavidin beads for 3 minutes. Beads were washed twice (300 µl each wash) with the reaction buffer. Pol I, ligase, NAD+ and dNTPs were added to perform the reactions. The products were loaded on a urea denaturing gel. A similar pausing pattern at the nick junction and a similar ligation in comparison to the Fig. 1D was observed (S7 Fig), suggesting that clamp loader complex has nothing to do with ligation.

**Nick translation coupled ligation assays on 3’ overhang template**

Further to extend our nick translation coupled ligation assay we generated a template annealing 67 bases, 19 bases and a new downstream 38 bases (GCGACTAATTTTAATGAGTGTGGGA TCCCTTATAATAG) oligonucleotide, as shown in S6A Fig. Therefore, the full-length product would be 67 nucleotides long if nick translation alone works. However, a 77 nucleotides long product is expected in the nick translation-coupled ligation reaction. The intensity of the 67 and 77 nucleotides band in the presence and absence of beta-clamp would demonstrate the role of beta clamp in ligation. When we used this template for the assay with Pol I and ligase, the 3’-overhang in the 77nt final product was degraded by the 3’-exonuclease activity of wild type Pol I with time (S6B Fig), suggesting that such a template is not an ideal one to calculate RLF in this regard. However, when we used exo^-^ Klenow or 3’-exo^-^ Pol I, the 77nt ligated product remained intact and RLF could be calculated at higher time points (S6C Fig and S6D Fig). These calculated RLFs were increased substantially in the presence of template-loaded beta clamp at each time points (S6E Fig).

**References**

55. Kozakov D, Beglov D, Bohnuud T, Mottarella SE, Xia B, Hall DR, et al. How good is automated protein docking? Proteins 2013;81: 2159-2166.

56. Georgescu RE, Kim SS, Yurjeva O, Kuriyan J, Kong XP, O’Donnell. Structure of a sliding clamp on DNA. Cell 2008;132: 43-54.

57. Yin Z, Wang Y, Whittell LR, Jergic S, Liu M, Harry E, et al. DNA replication is the target for the antibacterial effects of nonsteroidal anti-inflammatory drugs. Chem Biol. 2014;21: 481-487.

58. Emsley P, Lohkamp B, Scott WG, Cowtan K. Features and development of Coot. Acta Crystallogr. D. Biol. Crystallogr. 2010;66: 486-501.

59. Banks JL, Beard HS, Cao Y, Cho AE, Damm W, Farid R, et al. Integrated Modeling Program, Applied Chemical Theory (IMPACT). J Comput Chem. 2005;26: 1752-1780.

60. Bowers KJ, Chow E, Xu H, Dror RO, Eastwood MP, Gregersen BA, et al. Scalable algorithms for molecular dynamics simulations on commodity clusters.SC Conference, Proceedings of the ACM/IEEE, IEEE, 2006.

61. Shivakumar D, Williams J, Wu Y, Damm W, Shelley J, Sherman W. Prediction of Absolute Solvation Free Energies using Molecular Dynamics Free Energy Perturbation and the OPLS Force Field. J. Chem. Theory Comput. 2010;6: 1509-1519.

62. Jacobson MP, Pincus DL, Rapp CS, Day TJ, Honig B, Shaw DE,et al. A hierarchical approach to all-atom protein loop prediction. Proteins 2004;55: 351-367.

63. Sastry GM, Adzhigirey M, Day T, Annabhimoju R, Sherman W. Protein and ligand preparation: parameters, protocols, and influence on virtual screening enrichments. J Comput Aided Mol Des. 2013;27: 221-234.

64. The PyMOL Molecular Graphics System, Version 1.8 Schrödinger, LLC.
